# Taking Time: A Mixed Methods Study of Parkinson's Disease Caregiver Participation in Activities in Relation to Their Wellbeing

**DOI:** 10.1155/2020/7370810

**Published:** 2020-04-09

**Authors:** Lia Prado, Rebecca Hadley, Dawn Rose

**Affiliations:** ^1^Department of Psychology and Sport Sciences, University of Hertfordshire, College Lane, Hatfield, Hertfordshire AB10 9AB, UK; ^2^School of Music, Lucerne University of Applied Sciences and Arts, Zentralstrasse 18, Lucerne 6003, Switzerland

## Abstract

**Objectives:**

Although many studies have shown that psychosocial interventions, such as dance classes, can improve quality of life for people with Parkinson's disease (PD): few have addressed the role of, and potential benefits to, the caregivers in such activities. This mixed methods study explored the reasons for caregiver participation in a variety of activities and considered whether participation in, or abstention from these, affected the wellbeing of the caregivers.

**Method:**

Transcriptions of a focus group (two people with PD, two caregivers) and eight semistructured interviews (caregivers) were analysed using Grounded Theory (GT). To test the hypotheses derived from the GT, caregivers (*n* = 75) completed an online survey about activities they and the person they care for participated in, alongside the PDQ-Carer questionnaire, to establish the caregiver's levels of wellbeing.

**Results:**

Qualitative findings suggested that caregivers tried to find a balance between caring for the person with PD and participating in activities to attend to their own needs. Reasons for participating in activities for people with PD included being able to socialise in an empathetic safe space, alongside engaging in physical activity that provided some respite distraction, such as dancing with others to music. Reasons for not participating included generating time for oneself and increasing the independence of the person with PD. Quantitative results suggested that most of the participants' wellbeing was not compromised, although this was gendered: female caregivers reported lower wellbeing scores than male caregivers. Overall, 62% of caregivers participated in joint activities. Linear regression revealed a significant relationship between nonparticipation in daily activities and stress levels for female caregivers only, whereby the more independent the person with PD was, the lower the stress of the caregiver.

**Conclusion:**

This study suggests that caregivers of people with PD can find a healthy balance in terms of their own wellbeing by jointly participating in two-thirds of activities while ensuring the remaining third is time reserved for themselves.

## 1. Introduction

Parkinson's disease (PD) is a progressive neurological disorder caused by the loss of dopamine producing neurons in the substantia nigra [1]. The loss of these subcortical brain cells causes reduced dopamine levels in the striatum resulting in motor deficiency [2]. This presents behaviorally as movement impairments including tremor, postural instability, and freezing as common physical difficulties faced by people with PD [3]. However, nonmotor symptoms such as cognitive impairment and mood disorders, such as anxiety and depression, are also associated with PD [4].

As with any other chronic disease, individuals suffering from PD can see a significant decrease in their quality of life, and this can extend to affect those around them [5]. The caregivers of those with a progressive disorder such as PD can have their quality of life and wellbeing directly affected as the burden of care provision increases, reflecting the process of adapting to the new routines and changes that PD can bring. For example, caregivers can see their lifestyle change considerably, including the loss of social events, threats to self-esteem, and anxiety about the unpredictable future [6]. It is common for the caregivers of people with PD to be members of their family, and most probably, the spouse of the directly affected person [7]. Although individuals that suffer from PD and their caregivers have identified that the negative impact of this illness on their daily lives does not necessarily depend on the stage of the disease progression [8], the responsibilities of caring can affect their relationship as the caregivers' social life, and their careers can be compromised by the need to put their caring duties first [9]. The strains on caregivers tend to increase as the loss of mobility for those they care for impacts on their own freedom and the burden on caregivers can increase to the point where stress and additional problems with coping are perceptible [10, 11].

Several studies have shown that psychosocial interventions can be of benefit to people with PD in terms of health-related quality of life (QoL), a term that includes both physical and psychological wellbeing [12]. For example, activities as diverse as dancing, tai chi, yoga, strength exercises, music-making, and music therapy, as well as choirs have all been shown to have positive benefits for people with PD for both motor and nonmotor symptoms (see, e.g., [13–18, 19]). The term “psychosocial intervention” is commonly used to refer to any physical and/or social activity involving social factors that could influence individuals' minds or behaviors [20]. Although the social nature of the activities is rarely accounted for methodologically, one study investigating the effects of dance classes on mood reported improvements for people with PD as well as the age-matched controls who also participated [21]. This, and other studies focusing on dance classes have suggested that it remains an open and important question as to whether the observed changes in participants' in affective and physical states are due to the physical and/or partnered/group nature of the dancing, the affective properties of the music, and/ or the social connectedness enjoyed after the class during tea and biscuits sessions (see, e.g., [22–24]).

Whilst the evidence suggests that, in general, psychosocial interventions have positive effects on people suffering from chronic conditions, there are only a few studies that have included outcomes for caregivers specifically [25]. As one study identified, it is important to guide carers to obtain referrals to services, including counselling, support groups, and social workers [26]. However, there is a paucity of evidence in relation to caregivers of people with PD specifically, and in those that have approaches have focused on group or individual interventions with few integrated activities per se. For example, one study investigated the effects of in-home respite care on caregivers [27]. The program involved people with PD and their caregivers attending a daycare centre once per week for six consecutive weeks. Access to various psychological treatments with different health professionals was provided. The findings suggested that whilst people with PD showed improvements in speech, depression, and QoL in general, the caregivers did not show any change in measures of wellbeing [28].

Another study that also included people with PD and their caregivers focused on an education programme which comprised information about PD, a practical exercise using a self-monitoring tool, information about how to maintain health activities, stress management exercises, and advice for carers on how to prevent their responsibilities becoming a burden [29]. The findings showed a positive effect on caregivers' mood, but no significant effect on their quality of life or on their levels of depression. Two further studies focused solely on the caregivers. Marziali and Crossin [30] reported a qualitative study of an online support group which suggested that, by providing a space in which caregivers could share their concerns and learn how to cope with the difficulties and pressures of caregiving, positive benefits were realised experientially. Also published in 2005, Secker and Brown [31] reported the results on a study which showed that personalized sessions of Cognitive Behavioral Therapy reduced levels of distress for caregivers of people with PD.

Providing care for a loved one with a progressive disorder such as PD can have a direct impact on the wellbeing of the caregiver, and this is often operationalized in terms of Quality of Life (QoL). The concepts of QoL within the term “wellbeing” are difficult to define as they are open to interpretation [32]. Although there is no agreed definition of QoL, this study adopted the framework of the World Health Organization (WHO) QoL Group (1995), focusing on the perception that individuals have about their position in relation to the environment in which they live, their individual beliefs and values, and their individual life expectations. This perception of an individual's QoL can be extended to their wellbeing, which includes an individual's relationships with others, life purpose, understanding of the surrounding environment, self-regard, and individual growth and development [32].

Many caregivers accompany those they care for to activities that include a social aspect (i.e., psychosocial activities), though some do not. Although a few studies have considered the wellbeing of the caregivers of people with PD, it is not yet clear whether the suggested positive impact on wellbeing for caregivers is related to the intervention itself (for example, participating in the dancing), or the community of support provided by social contact with other caregivers and/or people with PD. Therefore, we first conducted a focus group and series of interviews with people with PD and their caregivers to try to understand what “wellbeing” meant to them, and how this related to whether or not they participated in activities with and without each other. This was followed by an online survey which specifically investigated QoL as operationalized in the Parkinson's Disease Questionnaire Parkinson's Disease Questionnaire for Carers (PDQ-Carer). This was developed specifically to evaluate the QoL of caregivers of people with Parkinson's disease by analysing Personal and Social Activities, Anxiety and Depression, Self-Care, and Stress. Using this process, we were able to test the generalizability of qualitative findings using quantitative data analysis. The study was ethically approved by the Ethical Committee with Delegated Authority at the School of Life Sciences at the University of Hertfordshire (Protocol LMS/PGT/UH/03287).

## 2. Materials and Methods

**Figure 1:**
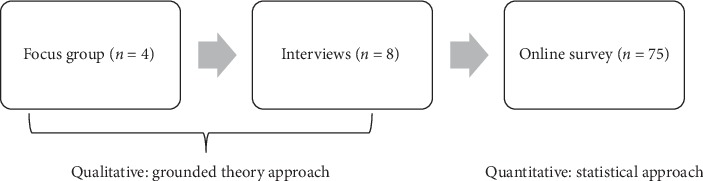
Schedule of mixed methods approach.

This study used a mixed-method approach (see Figure 1). The qualitative method used Grounded Theory analyses of focus groups and individual semistructured interviews. This method is outlined prior to the methods and materials used in the quantitative study (online survey), reflecting the inclusive and robust nature of the project development [34].

### 2.1. Qualitative Method

Grounded Theory (GT) is an appropriate qualitative methodological tool when there is a lack of research in the area under consideration. The approach is used to develop ideas and concepts with which to explore the subject in depth [35]. Descriptions of data are developed into themes, and the process is repeated until a conceptual structure (i.e., a model) is developed [36]. The researcher takes a reflexive path in the initial investigation, utilizing their expertise to build an information structure around the subject [37]. This method requires constant comparisons between items of data acquired in order to investigate each participant's experience in depth [38]. GT is an iterative process of data selection and coding, beginning with the choice of participants whose data is first described according to relevance with the research topic. Subsequent data acquisition contribute to the core themes until these are stabilized (point of saturation) whereby no further factors are found that contribute to the model [39, 40].

A focus group was conducted to identify questions to develop the semistructured questions for the individual interviews. This was undertaken as recommended by Parkinson's UK guidelines to ensure patient and public involvement (PPI) in the research process. The focus group and interviews were transcribed (Transcriptions can be made available on request) by the first author using the “Just the gist” method, in which only relevant parts of the interview are transcribed [41]. This method of transcription admits the possibility of losing some parts of the interview but is efficient for the purposes of this study because it makes the researcher focus on the areas that are most important in relation to the research question [42]. The transcriptions were then coded and analysed separately (using NVivo, Version 12, QSR Intl., 2018) by the first and second authors. The transcriptions were coded line by line using inductive reasoning to formulate themes. The research question started by considering wellbeing as a construct within the context of caring for people with Parkinson's disease. Attention then turned to participation in activities and how this might impact wellbeing (see Section 2.1.1). The two researchers compared codes and themes until an agreement was reached. These findings informed the online survey the themes generated suggested that the four factors of the PDQ-Carer (Personal and Social Activities, Anxiety and Depression, Self-Care, and Stress) would be appropriate for use as a quantitative measure.

#### 2.1.1. Focus Group Participants and Procedure

The process of undertaking a focus group to generate the questions used in the individual interviews ensured that directives for Public and Patient Involvement (PPI) were adhered to in terms of involving the people for whom the outcome matters the most in the research process. Initially, six participants were recruited for the focus group, though only four were finally able to participate. The participants (*n* = 4) included two sets of married couples, two people with PD (male), and two carers-givers (female) who were recruited from the Dance for Parkinson's group at the at the University of Hertfordshire. The mean age of participants was 77.25 years (SD = 2.62; range 75–80). Both participants with PD had been diagnosed with the disease for over two years, and both participated in more than one activity weekly. Their spouses acted as their carer-givers and participated with their partners in at least one of these weekly exercise classes. The focus group, conducted by the first author and moderated by the second author, took place in an accessible interview room and lasted approximately one hour. The interview was recorded using an Olympus digital voice recorder VN-8500PC.

Initially, five questions were posed to generate conversation during the focus group:

1. What does wellbeing mean to you?
2. What are the benefits of being well?
3. If you could have 10% more wellbeing, what would this enable you to do?
4. What are the benefits of attending the activities you choose to participate in?
5. Could you please say why you do or do not attend the classes with your partner?
6. These questions were retained for the semistructured interviews. Following the Focus Group, two further questions were added in order to probe the caregivers' feelings regarding being called a carer, or caregiver, and to consider which aspects had been hardest to cope with since the people they care for were diagnosed with PD.
7. How do you feel about being called a carer?
8. Could you please let me know what the most difficult thing that you have had to cope with since the PD diagnosis is?

#### 2.1.2. Individual Interview Participants and Procedure

A total of eight participants who were caregivers for people with Parkinson's were individually interviewed (*n* = four females). Participants were recruited from a range of local social activity groups including Parkinson's dancing, exercise, and support groups. The mean age of these participants was 69.33 years (SD = 7.86; range 57–79). All carer-givers confirmed that the people that they care for participated in at least one exercise activity.

Table 1 reports the carer-givers' characteristics.

The majority of the carer-givers were interviewed in person, though two were interviewed over the phone. The interviews lasted between 20 and 40 minutes and were recorded and transcribed as described in Section 2.1.

### 2.2. Quantitative Method

An online survey was devised to test the hypotheses generated during the qualitative study.

#### 2.2.1. Participants and Procedure

Participants were recruited via the Parkinson's UK Research Network. A total of 75 caregivers of people with Parkinson's disease (*n* = 16 male) completed the online survey. The mean age of the participants was 63.35 years (SD = 10.67; range 29–83). There was no statistically significant difference between the ages of the male and female caregivers (*p* > 0.8). The survey was made available online via the Qualtrics platform for psychological studies (Qualtrics, Provo, UT) and data were collected anonymously. Participants accessed the Participant Information Sheet at the beginning of the survey, then provided informed consent.

#### 2.2.2. Online Survey Development

After providing basic demographic data (age and sex), participants were asked to specify three activities that people they cared for participated in, whether or not they actively participated in these activities (compared to nonparticipation), and their reasons for attending or abstaining from participation in the described activities. The survey then delivered a questionnaire to investigate carers' quality of life, the Parkinson's Disease Questionnaire for Carers (PDQ-Carer; Professional version (29 items) Oxford University Innovation Limited, 2012). The PDQ-Carer questionnaire was developed specifically to evaluate the quality of life of carers of people with Parkinson's disease by analysing four different factors: Personal and Social Activities (12 items), Anxiety and Depression (6 items), Self-Care (5 items), and Stress (6 items). Each factor was calculated as follows: the sum of scores for each question in a factor is divided by 4 (maximum score per question) times the number of items in the factor then multiplied by 100. The 29 items are marked on a scale ranging from 0 (no problem at all) through 100 (the maximum level of problems). Scores within the range >60–100 suggest that the quality of life of the carer is seriously compromised [43].

## 3. Results

### 3.1. Qualitative

Grounded Theory (GT) analyses of the focus group and interview transcriptions were undertaken by the first and second authors to ensure agreement between codes and themes. These results are presented herein.

#### 3.1.1. Focus Group Results

**Figure 2:**
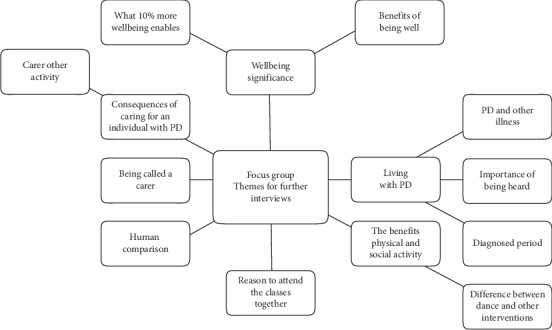
Model of factors derived from the focus group for inclusion in individual interviews.

Following GT analyses, seven themes (14 factors in total) were derived from the focus group.  Figure 2 displays a visualisation of the factors. As can be seen, the participants wanted the research to explore what wellbeing meant from a positive perspective (rather than, for example, in terms of critical theory). However, they also wanted the research to explore the nature of living with PD, the consequences of caring, and also the identification of carers in terms of what they are called and how they are compared to others. These factors were then included in the individual interview process to ensure PPI adherence for research design.

#### 3.1.2. Individual Interview Results

Following the focus group, eight individual interviews were completed using the questions identified in Section 2.1.1. The first and second authors analysed the transcriptions using Grounded Theory. In this instance, the factors identified during the focus group were used as initial thematic codes with secondary codes identified within this framework.  Table 2 shows the full list of codes and subcodes initially generated from the transcribed data by both researchers.

Following the discussion between the researchers regarding agreement on coding descriptions, these data were reduced to two main themes most relevant to the research question. (1) Reasons for accompanying individuals with PD to the interventions or not. (2) The benefits of participating in social and physical activities, and three subsidiary themes that arose from the process which are included to reflect the voice of the participants: (3) What being a carer for a person with Parkinson's disease is. (4) Carers' perceptions of living with Parkinson's disease. (5) The meaning of being well. The abbreviation PwP in Figures refers to people with Parkinson's disease. Direct quotations have been provided not only to support the analyses, but also to ensure the voice of the participants is appropriately represented in line with PPI recommendations. Commentary on subnodes in each theme will be conducted in a clockwise manner around each model.

#### 3.1.3. Theme 1: Carers' Reasons for Accompanying or Not Accompanying Individuals with PD to the Interventions

**Figure 3:**
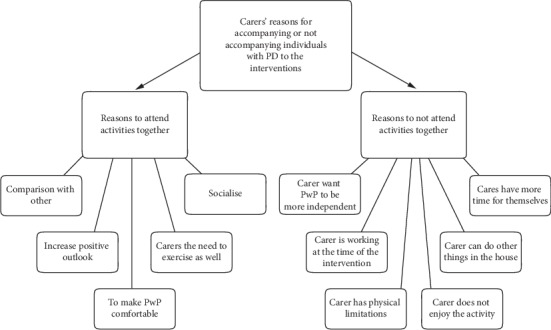
Model of Theme 1: carer's reasons for accompanying or not accompanying individuals with PD to the interventions.

As depicted in Figure 3, the caregivers clearly defined the reasons why they did (left side Figure 3) and did not (right side Figure 3) actively participate in activities with the person they care for, and the reasons for these decisions.

Firstly, considering reasons to attend, the caregivers explained that it was an opportunity for them to socialise, experience a positive outlook, and be accepted by being with other caregivers and people with PD who understood their shared circumstances, as these examples illustrate:

“We have got a fortunate Parkinson's group in the next village. They are a friendly huge range of people with Parkinson's at different stages, and they are also so cheerful and friendly, it becomes a community of things that you do together.” [P09]

“Yes, moving with music, she goes in a music Arena and three of us carers go into the kitchen and prepared refreshments and chat about life.” [P06]

“But to have a group of people that accept it and be able to participate and to contribute, they build hi's confidence, and that is good.” [P03]

It was also important to find a social space where the people with PD did not feel inhibited;

“She only goes out with people that we know well, so that she's comfortable sitting with them. And she is naturally so conscious about hiding her shaking hand or leg. It is all in one side at the moment. But those friends know, and obviously she doesn't want to talk about it, so yes the social side is definitely important.” [P07]

When talking about the dance classes specifically, some of the caregivers mentioned that the volunteers who came to help also provided comforting presence:

“In other activities, it is not the carers so much, but the volunteers, that are very sympathetic and very helpful, and very motivational, and over-using the English word, they are very nice. [P05]

For some, it was an opportunity to exercise *(“it makes me to exercise that I wouldn't do at home” P03*) and/or dance themselves, and this was especially important if this had been a part of their earlier lives together:

“I think that we dance together there, and we haven't done for long while, because he hasn't been fit to do anything. So at least that is good, because we used to dance a lot, and that is brilliant. [P02]

Ensuring that the people they cared for were comfortable was also important, and this ranged from just being there, to providing practical support:

“Yes, we need to do things together to enable her to do what she used to do.” [P09]

Another reason for attending together was about generating a positive outlook, even if it was a short distraction from reality, as two caregivers related:

“The benefits for me is to see that he's enjoying it, and when I am at the class, you can forget everything else.” [P04]

“We have a laugh and we enjoy it, because I don't always get it right! So we have a laugh as well with silly little things like that. It is nice.” [P08]

Attending the same activities also served another purpose, being able to self-monitor and compare disease progression between the people with PD. As several caregivers described when talking about the people they care for and also themselves:

“You know what, he likes to see everybody else, he watches everybody else intently, and he likes to know their age.” [P08]

“It is always good compare with someone else, it is a natural human process” [P05]

“Seeing all the different stages - more how people are managing and coping with the difficulties that they have really, it's nice to know that they're around.” [P09]

Others explained they simply stayed because the intervention period is not long enough for carers to do something for themselves:

“Well there is not enough time to go somewhere else or do something else anyway.” [P07]

Understanding the reasons that carers do not participate in the activity is as important as the reasons others do, and among the caregivers interviewed, these reasons varied. For some it was because they were working at that time or had their own physical limitations that prevented participation in that type of activity. For others it was simply because it was not something they wanted to do:

“I never enjoyed dancing, number one, probably this is the first thing that comes to mind.” [P05]

The caregivers sometimes took the opportunity to engender more independence in the person they cared for:

“I'm not really sure how much he enjoys it, but I go to an art class when he does that and he understands that I go to my art class, so he will go off to the day centre.” [P04]

This creating of space for the caregivers was one of the main reasons for not attending activities with those they cared for. The caregivers made an explicit choice to make time for themselves to do something else, whether that was getting a chance to put things straight in their homes, or time “off” from being a caregiver and to be able to think about themselves for change as these examples illustrate:

“Life is busy and there are other things that I would like to do and get done, which to my mind are more important than exercising, but it's probably right that I need some exercise, but I do get more exercise from the work that I do at home.” [P03]

“This is not the real reason, I mean I don't like dancing so, but I need some time for myself […] I like to have my own space […] so now when she goes out I feel that I can just relax and that is why I don't go to the dance.” [P09]

“I just need to think about myself a bit and not always be worrying, so now when she goes out I feel that I can just relax and that is why I don't go to the dance. So to just have a morning that I don't have to think about that it is actually quite nice. It sounds very selfish.” [P06]

Whether they attended and/or actively participated or not in the activities, the caregivers provided insights regarding the potential benefits they perceived as identified in the next theme.

#### 3.1.4. Theme 2: The Benefits of Participating in Social and Physical Activities

**Figure 4:**
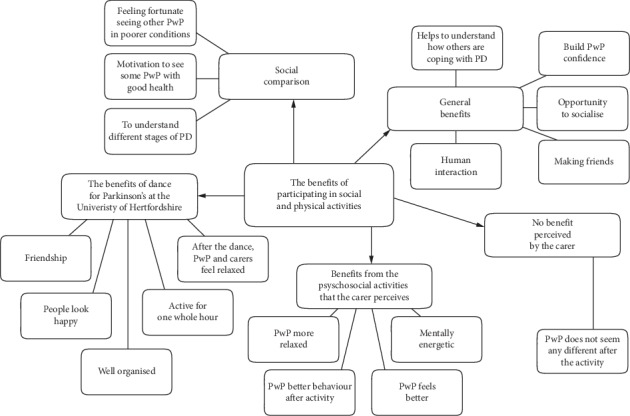
Model of Theme 2: the benefits of participating in social and physical activities.

As shown in Figure 4, the main benefits of participating in social and physical activities were also identified as a reason for attending (Section 3.1.3.). Here we focus on further insights provided by the caregivers from their perspective in relation to the potential benefits participation could bring. For example, the caregivers noted that there were two types of physical benefit they observed; one being a positive effect of exercise and the other being the inducement of fatigue, as the following excerpts illustrate:

“It is good, and it's good because if she didn't exercise, because of the Parkinson's, her muscles would disappeared in life would become more difficult, so it is keeping her active. You can say that for me it's like a dog food, it prolongs active life.” [P06]

“Immediately afterwards we are slightly shattered, but I think probably we do feel better than when we don't do it, because sometimes you forget – something comes up and you don't do it. I think it is more that we notice when we don't do it! It is like with the walking, we noticed more when we not have been walking. We are more sluggish, whereas outside makes you feel brighter at the end.” [P12]

As the previous excerpt suggested, others also noted that the physical exertion and challenge could also lead to positive psychological benefits:

“He tries so hard to be able keep up with everybody, and I like it because he enjoys it and when he comes back he's tired he feels like he has done something.” [P08]

“He's more relaxed I think, he's quite jubilant, he just has got more energy, although he is tired, mentally he's more energetic.” [P09]

“Yeah it certainly doesn't do any harm, I suppose you can say she comes back feeling better and therefore because she feels better, she behaves better if you like, a bit more relaxed.” [P10]

“But also, it boosts her confidence. That she can interact with people as a level where there is joint sympathy… empathy with one another.” [P06]

Two caregivers who did not attend activities with those they cared for stated that they did not observe any noticeable benefits following a physical and social intervention:

“I'm not sure physically he gets benefit out of it.” [P03]

“For me she doesn't look different.” [P08]

For one of these caregivers, in contrast to others suggested that the activities provided a place to go to compare the progression of PD; this was actually seen as a problem and a potential barrier to participation:

“She was afraid that what you was going to see how she was going to see later on in life, possibly.” [P03]

The next theme focuses on the concept of wellbeing as experienced by the caregivers of people with PD.

#### 3.1.5. Theme 3: What Being a Caregiver for a Person with Parkinson's Disease Is

**Figure 5:**
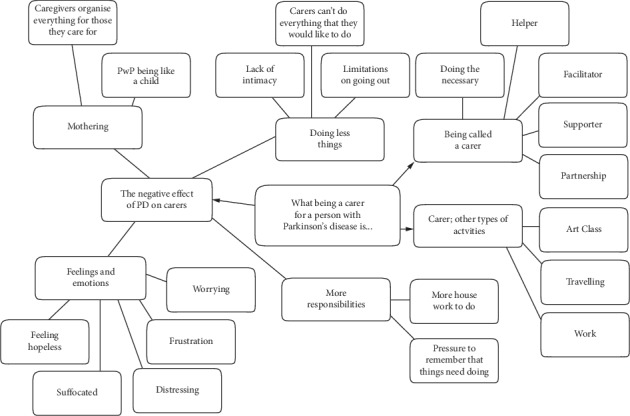
Model of theme 3: what being a carer for a person with Parkinson's disease is from the perspective of caregivers.

In relation to this question, generated during the focus group stage, three main subnodes were generated: Being called a carer, Carer other activities, and The negative effect of PD on Carers (see Figure 5).

In general, it was considered significant to be recognised as a carer or caregiver, rather than simply a partner (who might just be someone you lived with). Being called a carer was not limited to helping people with PD with their physical needs, but also provide emotional support as these examples indicate:

“Partner does not mean anything; carer does. It implies that the other person needs help.” [P01]

“I don't feel like a carer. I am a carer, but it's not so much hands on, it is more providing moral support I guess.” [P10]

Caring for a spouse, not only with regard to illness, was seen by some caregivers as included in their commitments of matrimony, which in turn was perceived as a strength in a relationship:

“Some people think the term ‘carer' diminishes the relationship, but actually this enhances it. I think it is almost that people think you're downgrading from lover to carer but it's not.” [P05]

Overall, identifying, or being called a “carer” was seen as doing something that was necessary and part of being a partnership, and sharing the burden. Caring also incorporated being a supporter, a helper, and a facilitator for the person being cared for, though getting the balance right often took time to negotiate as this example illustrates;

“We need to do things together to enable her to do what she used to do, though I have to stop myself being too helpful, if you see what I mean.” [P09]

Although it was evident that caring for their significant other was the participants' main occupation, the caregivers also recognised that the care they provided had to be done within the framework of their own lives. For example, some carers were still working, whilst others liked travelling, or had other individual hobbies (e.g., Art Classes). However, these were described as being “other activities” suggests some sense of conflict in terms of priorities between their own needs and the needs of the person they care for.

The main factor in this theme related to the negative effect of PD on the caregivers themselves, and in particular, how this affected their personal wellbeing. Firstly, the caregivers found they had to take on more responsibilities in the home in terms of practical functions such as washing up, to taking care of bills, but also remembering all the daily aspects of their lives, as one participant explained:

“I have got to remember everything, for both of us. That is the problem. So you've always got to be on the board keeping an eye on things.” [P02]

Caregivers expressed that their feelings and emotions were negatively affected, including worrying about the person they cared for and themselves. Caregivers spoke about the frustrations they experienced, feeling that they could not do more, for example, to reduce the pain their loved one experienced, and/or live the lives they had expected to enjoy either themselves or together:

“My problem is I don't know what to do, how can I help her in this situation” [P03]

“I get the impression that the attempted help I seek to give doesn't always help, so it's my frustration and no doubt for X as well.” [P06]

Although caregivers explained that they tried to stay positive, they also reported high levels of distress to these imposed changes, as well as feelings of hopelessness and suffocation as this exemplar conveys:

“You have to be 100% positive, if you can… I slowly see that the man that I love disappearing from me. …how claustrophobic it feels; how you want to scream sometimes!” [P02]

One way in which participants expressed the negative effects of PD was the way in which their relationship had changed; for example, instead of being equal partners, some felt that they had needed to adopt a mothering role. This had a functional aspect in terms of having to organise everything, but also a sacrificial aspect as participant three explained: “My life is for their benefit.”

This change in relationship status also brought with it unwelcome physical limitations on available intimacy as one participant explained:

“…he has been impotent for quite long while, and this is something that we live with. Is not something that we tell many people, but that is a special thing in the relationship.” [P01]

More generally, the caregivers simply felt that they were able to do fewer things than they used to do, especially limiting their ability to go out.

“Oh yes, I used to do lots of things. I hate routine, so this for me is killing, because it's so boring” [P04]

Ultimately, the main feeling that the caregivers expressed in relation to how PD had affected them was a sense of resignation to their role as caregivers within the relationship, as expressed here:

“This is what you do. You just accept it”. [P06]

Although a sense of spousal duty often seemed to underpin the long-term commitment to providing care, there was palpable awareness that the burdens on the caregiver would grow as the disease progressed. Consequently, the next theme explores in more detail the nature of living with PD from the caregivers perspective.

#### 3.1.6. Theme 4: Carer Perceptions of Living with Parkinson's Disease

**Figure 6:**
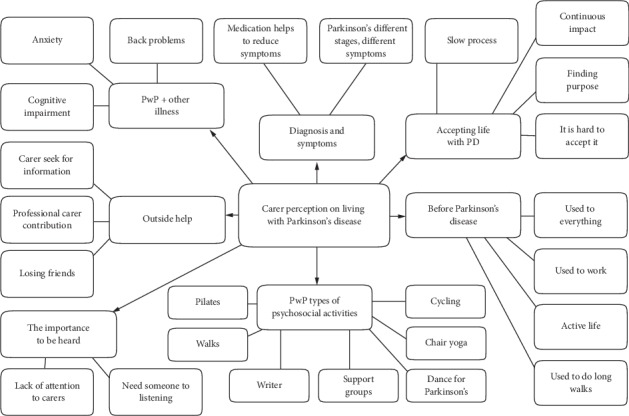
Model of theme 4: carer perception on living with Parkinson's disease.

GT analyses resulted in seven major subnodes in this theme, as depicted in Figure 6. The caregivers spoke about their experiences of symptoms and diagnosis and their struggles with accepting the situation. They also reminisced about what life was like before PD. As discussions turned to their choice of activities and interventions, the caregivers expressed how they often felt their needs were secondary to the person they cared for, despite the enormous impact PD had on their own careers and lives.

All participants were fully aware of their partners' symptom history and development, how the diagnosis had been completed, and of their daily medication regimes (i.e., the timing of which drug was taken when) for best effect.

In terms of acceptance of living with PD, the caregivers mentioned the physical and psychological aspects of the impact of PD as these examples relate:

“It the cognitive side that causes him an awful lot of worry. He worries all the while. Like if I say I'm back in five minutes and he sees that I'm not there, he starts panicking, you know.” [P02]

“His speech is quite poor at the moment. I can't hear straight away a lot what he says, and actually that is quite strain because he gets fed up with me asking him to repeat himself. It's hard to go out because he had really bad dyskinesia. You know he couldn't really sit on chair for Match of the day. By the end of it, he was nearly on the floor!” [P06]

“She doesn't like going in the tube because that increases her anxiety, there are a lot external factors that trigger the anxiety, this is another thing that I had to learn to understand, because of course doesn't make any difference to me.” [P10]

The main impact on the caregivers was the shock of the sudden change in their lifestyles, as one participant explained:

“Everything you do reminds you of those things that you cannot do anymore. So that is why I remember the date [of diagnosis]. My life stopped. Everything I did stopped.” [P02]

This seemed to be exacerbated with the sense of loss associated with experiencing the deterioration of the person they cared for:

“Actually it is seeing your husband deteriorating, you know it is quite hard to accept it, particularly at first, he's changing and it is the hard thing I think that I had to come to terms with.” [P09]

Some caregivers also experienced a sense of loss for all the things they used to do but had been able to adapt similar types of activities to compensate. For example, one couple used to enjoy eating out at restaurants, and although this was becoming increasingly difficult, the help of friends had been enlisted to facilitate all-round enjoyment:

“We went out for dinner on Saturday. X comes with us; he loves it. We have two friends that we go out with, and they understand the situation, so they're very good. They are very inclusive with X and they make sure he is joining in, which is brilliant”. [P02]

However, many of the caregivers spoke about the loss of friendships as part of living with PD.

“Some people heard about his Parkinson's, promised support, but in the actual fact they then disappeared. Whether they were embarrassed, or didn't know what to say, or didn't know how to cope, we don't know. Some people almost became dismissive, perhaps critical, who knows.” [P03]

The loss of support seemed to especially impact the caregiver, increasing feelings of both anger and isolation:

“I think the interesting thing is that you lose a lot of friends, because actually they don't understand it at all. I have got one or two that are very understanding, but they are understanding because they have done some caring for themselves, so they understand the stress that is put on your life. And people don't talk to him, they don't talk to him, that makes me feel really angry, because they are embarrassed and they don't want to say, the only thing they have to say to him are you alright? This doesn't hurt anyone but they don't do it. [P07]

Similarly, the lack of continuity for PD and PD Carer support was problematic:

“You have all these people coming to your front door, and they sit on your sofa with a piece of paper and a pen, and they all asked you the same questions and they go away and you never see them again!” [P04]

However, this is where specialised support groups and networks became important. As participant six explained:

“We both go to Parkinson's UK. I also committed to the support group because we got Parkinson's. It is something that we both need to cope with and we need people who understand what we are going through – and the Parkinson's and support group does that. You do that on the Friday here, participating, so it is all good.” [P06]

In addition to PD, all caregivers reported that the people they care for had other illnesses, as well as health issues they had suffered themselves (such as back problems), and together these could overload their ability to deal with their situation. Considering all these difficulties reported by carers, one positive outcome was that all caregivers confirmed the people with PD that they care for had become involved in at least two psychosocial activities since being diagnosed. The next theme considers what 'wellbeing' means from the perspective of caregivers of people with PD.

#### 3.1.7. Theme 5: The Meaning of Being Well from the Perspective of Caregivers of People with PD

**Figure 7:**
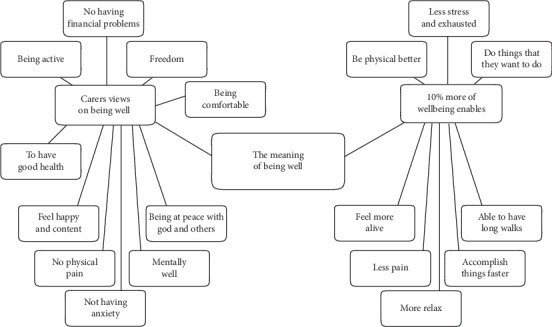
Model of theme 5: the meaning of being well for caregivers of people with Parkinson's disease.

During the focus group, a discussion had developed in relation to what the concept of being well meant for people who care for those with a degenerative disease; in these cases, Parkinson's. During the interviews, the caregivers explained what being well meant for them and considered the concept further by exploring what 10% more wellbeing would look like for them (see Figure 7). The following section relates the details of these two aspects of wellbeing for the caregivers of people with PD.

With regard to wellbeing in general, the caregivers expressed that this would mean

“You would feel happy and content, and ok to be alive.“ [P07]

“To me, wellbeing means being able to do all I used to do.” [P01]

“Comfortable. When someone says wellbeing, I imagine being comfortable in what you're doing and how you feel.” [P11]

“It is a combination of physical, mental and psychological – and being comfortable with what you are. On the other hand, everyone has got stress in their lives, everyone has physical pain in their lives. You cannot be well all of the time, and one person's view of being well is not another's.” [P08]

“Wellbeing means to me feeling that my brain is alive and I can be active.” [P03]

For some carers the understanding that PD will be a constant part of their lives leads them to believe that their wellbeing will no longer be able to reach the 100% mark:

“I think, before PD I would say the level of wellbeing was 100%, with usual fluctuations. Now with PD in the household it is almost 75% type level, there is always this underlying thing, like it's always going to be something, PD is always going to affect something. We will never going to get back to that 100% carefree feeling to do whatever we want to do.” [P10]

When asking carers to explain what 10% more wellbeing would enable them to do, the majority said that they would feel less fatigued and be physically able to do more:

“I think I would be able accomplish things faster, than the things I do now. It could enable me to do what I do without feeling half dead, because I have to push myself hard to do what I do.” [P06]

Psychological wellbeing would mean suffering less stress and anxiety:

“I suffered very badly of anxiety for a long time, for about 9 months to a year before I got better. So wellbeing is not feeling like that. Because it was really awful and I never felt like that before. So wellbeing is feeling fairly content, and not feeling worried all the time, to be more relaxed, because he is quite advanced with Parkinson's, it is quite stressful at times.” [P06]

Finally, being well would enable carers to feel a greater sense of freedom:

“Well, if the wellbeing was 100%, yes it will be a feeling to be completely free and not having to worry about it. Yes, it is a freedom thing.” [P07]

The insights from the qualitative data analyses suggested that the caregivers' quality of life could be compromised, affecting their sense of wellbeing. However, it was not clear whether actively participating in psychosocial activities was experienced as more or less beneficial in comparison to taking time for oneself. Furthermore, it seemed that the social aspects of participation seemed important for those who did attend, potentially being more beneficial to their wellbeing in comparison to taking part in the physical aspects of activities and interventions. Consequently, a quantitative study was undertaken to test these hypotheses specifically:

  H_1_: Higher levels of joint participation in psychosocial activities will increase wellbeing for the caregivers of people with Parkinson's
  H_2_: The social aspects of activities provide more benefit to wellbeing compared to physical activities, home-based entertainment, over and above daily (functional) activities

The following section addresses these questions.

### 3.2. Quantitative Results

#### 3.2.1. Descriptive

Overall, the 75 participants reported that the people with Parkinson's they cared for took part in 195 activities. The caregivers actively participated in 63% of these activities in total.

Participants were asked to describe up to three activities that individuals with PD they care for are involved in, whether the caregivers actively participated or not, and why. All reported activities were grouped according to their nature, resulting in four categories; physical, social, home-based entertainment, or daily activities. Physical activities included all types of physical exercise, such as yoga, cycling, dance, walking, seated exercise, swimming, physiotherapy, and table tennis. Social activities included going to the cinema, going to church, meeting with friends, attending Parkinson's-related meetings, going to concerts or the theatre, and any type of group therapy. Home-based entertainment included board games, online games, TV, Sudoku, and crosswords. Daily activities included all home chores such as gardening, cooking, shopping, house maintenance, and looking after the kids or grandchildren.

Table 3 reports how many caregivers participated in the four categories of activities for the whole group and by sex and depicts the relative percentage (compared to the whole sample by sex) of the active participation of male and female caregivers in each of the four activity categories.

#### 3.2.2. Parkinson's Disease Questionnaire: Quality of Life (QoL) of Caregivers (PDQ-Carer; [43]

Table 4 presents the mean, standard deviation, and level of compromised quality of life (QoL) for the whole sample and by Sex. The overall results suggest that in this sample, on average, the caregivers for people with PD's were predominantly within the noncompromised margin in terms of their QoL. However, as between a 5^th^ and a half of individuals' scores suggested their QoL was compromised in each of the four categories, these data are also reported in Table 4. To investigate whether this was gendered, the percentage of compromised QoL relative to sex in the whole sample was computed (also see Table 4). Due to uneven group sizes, *t*-tests were used to investigate differences between Sex for all PDQ-Carer factors.

For Personal and Social Activities, a statistically significant difference between sex was revealed, *t*(73) = 3.78, *p* < 0.01. Male caregivers reported better levels of personal and social activities than female caregivers of people; *Mean Difference* = 19.72, *CI* = 9.31–30.13.

For Anxiety and Depression, a statistically significant difference between sex was revealed, *t*(73) = 2.40, *p* < 0.05. Male caregivers reported less anxiety and depression than female caregivers; *Mean Difference* = 14.75, *CI* = 2.48–27.02.

A statistically significant difference between sex was also revealed for Self-Care, *t*(73) = 2.10, *p* < 0.05. Male caregivers reported higher levels of self-care than female caregivers; *Mean Difference* = 14.62, *CI* = 0.74–28.50.

For Stress, a statistically significant difference between sex was also revealed, *t*(73) = 2.69, *p* < 0.01. Male caregivers reported lower levels of stress than female caregivers; *Mean Difference* = 19.55, *CI* = 5.08–34.02.

In order to test whether either the level of participation or the type of activity was related to the caregivers QoL (in terms of the four factors of the PDQ-Carer scores), simple linear regression analyses were undertaken between the total amount of participation and wellbeing scores and amount of participation and nonparticipation between the four types of activities as predictor variables for the wellbeing scores. However, no significant relationships were found between the level or type of participation and the QoL scores overall.

## 4. Discussion

This mixed methods study investigated which type of activities caregivers do and do not attend with the people with Parkinson's that they care for, and the reasons for these choices in terms of perceived benefits for the caregivers. Both qualitative and quantitative findings showed that caregivers participated in activities and interventions with the person they care for the majority of the time (63%). A range of activities was reported and these were categorized as Physical (97% caregiver participation), Social (48% caregiver participation), Home-based Entertainment (20% caregiver participation), and Daily activities (30% caregiver participation).

The findings from the qualitative study (i.e., Grounded Theory) suggested two working hypotheses: (1) that higher levels of joint participation in psychosocial activities will increase wellbeing for the caregivers of people with Parkinson's, and (2) that the social aspects of activities provide more benefit to wellbeing compared to physical activities and home-based entertainment, over and above daily activities. However, statistical analyses of the survey did not support either prediction as no relationship was found between higher levels of joint participation in activities and better wellbeing scores, and similarly, the type of activity was not associated with particular benefits in terms of QoL. As indicated in the quantitative study, in which the majority of the caregiver's wellbeing was not compromised, the caregivers tended to split their time by participating in activities with the person they care for two-thirds of the time and reserving one-third of their time to provide for their own wellbeing (i.e., protected time). The need for balance identified in the current study was also demonstrated in a study by Roland and Chappell [48] that examined the relationship between caregiving and symptoms/strains and outcomes to develop typologies of informal caregiving across several different diseases, including PD with dementia. The authors identified that motor complications, symptomatic of PD, lead to the greatest worry and increased caregiver strains due to the hypervigilance needed to ensure the care receiver remained safe. Thus, the authors emphasise the increased need for caregivers to take breaks, not only to seek relief from overseeing the physical safety of the care recipient but also to obtain social support. The results of the current study suggest that caregivers of PwP are able to find a balance between taking time for themselves and engaging in activities that provide social support, such as dance classes, as indicated by the qualitative data. While it is not known in the current study how many caregivers were caring for someone with Parkinson's and dementia, mild cognitive impairment is common and it is estimated that between 30 and 40% of people with PD will eventually be diagnosed with clinically significant dementia. Consequently, the null hypotheses suggest that finding the right balance of participation in activities appropriate in each circumstance supports the wellbeing of the caregiver in general. However, the quantitative data also showed that female caregivers reported significantly lower wellbeing than male caregivers across all four factors of QoL, including personal and social activities, anxiety and depression, self-care, and stress.

Although other studies of wellbeing in caregivers for people with Parkinson's also have reported more female than male participants [27, 44], these studies did not analyse their findings according to gender. Whilst the gendering of the negative impact of the burden of care may appear initially to align with the higher prevalence of Parkinson's in men than women [45], in fact, the weighted QoL scores showed that half the female caregivers were at risk of having their wellbeing compromised in comparison to a third of the male caregivers (Table 4). This finding is supported by previous studies such as Morley et al. [46], which used the same measure of QoL and also reported significantly lower QoL in female compared to male caregivers. The authors suggested that this may be due to differences in coping strategies whereby men tend to be more problem focused and women are more emotion focused based on a mixed methods study by Almberg, Grafström, and Winblad [47]. However, in that study, participants were grouped according to the risk of burn out, and as the high-risk group contained 15 females, but only 2 male participants, in comparison to the nonburn-out group which was evenly balanced, it is difficult to draw any firm conclusions in that instance.

Although not specific to Parkinson's care, Pinquart and Sorensen [48] provided a robust meta-analysis of 200 studies pertaining to gender differences in caregiver stressors. Whilst their data confirmed that the majority of care is provided by wives and daughters (71.5%), they questioned the theoretical frameworks of gender-role socialization (Gilligan, 182), gender-role expectation [49] as previous studies (e.g., [50, 51]) had reported inconsistent results, probably because the differences reported, though statistically significant, were generally small. The conclusions of their meta-analysis were that there are four predictors of caregiver's stressors: burden experienced, level depression, the amount of care provided, and the quality of relationship with the care receiver. Furthermore, gender differences are apparent because women experience more caregiving stressors, but this is not stereotypical as although husbands and wives' experiences appear to be similar, sons and daughters are different, but this is subject to social change according to more recent studies. The authors recommended that interventions for caregiver wellbeing should attend to the specifics of the individual situation with a focus on providing help with activities of daily living.

This is in line with the recent study by Park and colleagues [52] who found female caregivers for people with dementia including Parkinson's-related dementia, who were living with the care recipient “had little opportunity for respite from caregiving, which increased their reports of feeling burdened” (p. 269). Lethin and colleagues [53] also noted that the wellbeing of male caregivers was better than female caregivers in their study of dementia care. However, they were able to track important details, which showed that these caregivers were predominantly adult male children who were not living with the person with dementia. It is a limitation of this study that such demographic data was not collected. It is a further limitation of this study that data relating to the stage of Parkinson's disease was not gathered. For example, the collection of Hoehn and Yahr [54] disease stage data may have helped elucidate some differences in caregiver wellbeing associated with disease progression, as found in Carter et al. [27]. In that study (in which 70% of participants were female), though they concluded that although in general the burden of care increased over time, they cautioned clinicians to use the stage of disease progression “only as an index of suspicion” (p. 26) as their study showed large variability in caregiver strain across all stages of Parkinson's. The authors suggested providing more respite care for the partner and family caregivers could reduce subjective strain. Although the nature of self-report in online surveys means data cannot be verified, future studies should include disease duration as a minimal marker pertaining to disease progression. Furthermore, whilst using the Hoehn and Yahr may not be possible without clinical experience, the Schwab and England Activities of Daily Living Scale [55] could feasibly be inserted into an online study to enable differentiation between participants at a basic level.

Although no direct link was revealed between the amount or type of activity as predictors of wellbeing using statistical analysis in this study, the qualitative findings provided a rich source of data regarding the nuances of how different activities may support caregivers in certain ways. The themes uncovered by the application of grounded theory helped to explain what it is to be a carer for people with PD, although some participants did not consider themselves to be caregivers in the physical sense, as the person they care for is very able. However, even then, the caregivers reported giving moral support where no physical aid was needed. The notion of moral support was particularly interesting in relation to one of the reasons given for attending activities, social comparison. In this instance, caregivers reported that both they and the person they care for took the opportunity to compare themselves to other people at various stages of disease progression. Buunk et al. [56] suggest that social comparison can have a positive side but also a negative side, and this was also the case herein. Some caregivers reported that seeing other people experiencing worse conditions (in terms of disability) helped them to feel more content with their current situation. However, other caregivers specifically stated the reason they did not attend activities for people with PD was to avoid seeing what they may face in the future.

Most caregivers related that they enjoyed participating in the physical activities and felt that taking part had a positive impact on their own wellbeing (providing an opportunity to exercise) as well as for the person with PD. As found in Tanaka et al. [57], this seemed to be related to the amount of independence the person with PD had and how much autonomy the caregiver felt they needed. Indeed, some caregivers explained that they preferred to do something else while the person with PD they care for was busy participating in an activity, For example, the caregivers could take this time to do something for themselves (e.g., art classes), see friends, or simply relax at home by themselves, or even just “get things done,” such as taking the opportunity to do shopping or cleaning. Certainly, whether jointly participating or not, the caregivers identified that the benefit they experienced from the person with PD taking part in an activity reflected benefits for them indirectly, such as improved mood, more confidence and positive communications, and/or a beneficial type of fatigue that enabled physical rest which in turn was beneficial for the caregiver.

Some studies have emphasised the negative impact on the social aspects of caregivers' lives (e.g., [59]). In the present study, caregivers also mentioned how difficult living with PD is in terms of their social life with some also reporting losing friends because of PD. Due to the nature of recruitment, this study was able to investigate the specific benefits of psychosocial activities involving a group of people, such as Dance for Parkinson's, support groups, and other group activities that have the distinct benefit of combining physical and social activities at the same time. The social element was one of the main benefits mentioned by caregivers in this study. Although not formal respite care, it seemed that the caregivers could enjoy the dancing as a physical release and also see the enjoyment for the person they care for. Moreover, the social aspect following the dancing, or “tea and biscuits” effect, enabled a safe, empathic space whereby people experiencing the same challenges could support each other either through simply listening and understanding, or providing practical help such as transport and disability aid solutions.

Goldsworthy and Knowles [60] suggested that one of the stressors predictors in carers for people with PD is the lack of social support. The need to identify interventions that benefit caregivers for people with PD has been a challenge (e.g., Greenwell et al., 2015; [25]). This current study showed that a majority (63%) of caregivers participate in at least one physical or social activity with the person with PD they care for. However, it should be noted that online recruitment failed to identify whether the respondents were partners, family members, or professional caregivers. Future studies should try to differentiate between types of caregivers and the activities they participate in providing a fuller picture of which areas are most beneficial for the caregiver's wellbeing. Similarly, studies could take a dyadic approach to understand how the wellbeing of both the caregiver and the care recipient influence each other.

## 5. Conclusion

This study tracks the quantity of caregiver participation in activities for and with the people with PD. They care for using a mixed methods approach. Grounded theory analyses of in-depth interviews with caregivers suggested that taking part in psychosocial activities provided both the caregiver and the care recipient with a supportive social network in an empathetic safe space. Although a follow-up quantitative study failed to link the type or amount of participatory activity with specific benefits to caregiver wellbeing, it did, however, suggest QoL was lower in female compared to male caregivers of people with PD. Overall the findings of this study suggest that a good balance for wellbeing can be found by ensuring that the caregivers reserve one-third of their time for attending to their own needs. As the rapid increase of long-term conditions causes further strain on the already overburdened and underfunded health system, providing sustainable community solutions in partnership between healthcare services and the social sector is a necessary step [61]. As such, psychosocial activities such as dance for Parkinson's should be further explored in relation to the provision of informal respite care with bidirectional benefits for both the caregiver and the people with Parkinson's.

## Figures and Tables

**Table 1:** Summary of caregiver characteristics from individual interviews.

Carer ID	Years since PD diagnosis	Number of activities	Activity example 1	Activity example 2	Reasons not to participate	Reasons to participate
P01	2.5	2	Dance	Exercise	When PD individual goes to exercise, carer can go to art class	When PD individual goes to dance, carer can enjoy this as well as they are dance partners

P06	9	3+	Dance	Moving with music	When PD individual goes to dance, carer can do other things. Carer also has physical limitations	When PD individual goes to moving with music, carer can go and enjoy the company of other carers as they prepare the refreshments

P07	4	2	Music	Day care exercise	When PD individual goes to the day care, carer can go to art class	When PD individual goes to music herts, carer needs to stay as there is not enough time to go somewhere else

P08	5	3+	Dance	Walks	Carer does not like dancing	They do walk sometimes together

P09	17	2	Dance	Cycling	Carer does not like dancing, and enjoys having time to do other things	Carer does not cycle, and enjoys having time to do other things at home

P10	5	2	Swimming	Walks	Carer works	They do walk together sometimes.

P11	8	2	Dance	Physio exercises at home	Carer needs to help PD individual (professional carer)	Carer needs to help PD individual (professional carer)

P12	0.8	2	Online chair yoga	Walks		Carer finds the need to exercise as well. They walk their dogs everyday together

**Table 2:** Major and subnodes following grounded theory analyses of eight interviews with caregivers of people with PD.

Major themes and subcodes	Researcher 1 N comment/s coded	Researcher 2 N comment/s coded
The negative effects of PD on carers (total)		
Doing fewer things in general	14	19
Difficulties getting out	4	5
Less travel and holidays	2	2
Loss of intimacy	1	1
Increased responsibilities	3	4
Adapting to new routines	5	4
Mothering/being “overly” helpful	6	5
Loss of freedom	7	8
Sense of suffocation/claustrophobia	2	3
Feelings and emotions		
Depression	2	2
Anxiety	3	2
Stress	4	4
Guilt/Selfishness	3	4
Associated with religion	1	1
Associated with love and marriage	3	4
Associated with sense of duty and sacrifice	3	3
Loneliness/lack of understanding	3	3
Loss of friends	3	4
Carers' perceptions of living with PD		
What being a carer means for caregivers of PwP	8	7
Accepting life with PD	5	6
Life before PD (reminiscence/change/loss)	6	5
Diagnosis and symptoms	9	11
Experienced with outside help for people with PD	3	2
Issues related to cognitive impairment in addition to PD	8	6
Issues related to caregiver ailments	1	2
Number of activities discussed	12	12
Exercise	4	4
Dance for Parkinson's/music group	6	6
Enjoyment of music	3	3
Support group	2	2
Total beneficial reasons for accompanying PD individuals to the activities		
Motivation to do “something,” rather than nothing	2	3
Socialising	9	6
Shared understanding in safe space	4	4
Increased positivity	5	4
Seeing benefit for person they care for (mood, confidence, independence)	6	5
Physical (increased energy/fitness)	4	4
Increased fatigue	3	3
Comparison with other people with PD (disease progression)	5	5
No benefit perceived by carers	3	2
Only attend because not enough time to do something else	2	3
Barriers to participation		
Physical deterioration (including speech)	7	8
Fatigue/Apathy	1	2
Discomfort at facing future impact of PD	2	2
Does not like the activity	4	3
Benefits to caregiver by not attending the activities		
Freedom from PD/distraction from reality	3	4
Time to self	4	3
Different interests	2	1
Increased independence of person with PD	2	3
The meaning of being well for caregivers of person with PD		
Contentment	5	6
Physical	6	6
Lack of pain	2	3
Less fatigue	3	2
Psychological	7	5
Less depressed	1	1
Less anxious/worry about future	3	4
Less stressed	4	3
Autonomy	3	4
Financial	1	1
Reminiscence for freedom	2	3

**Table 3:** Types of Activities and level of caregiver participation^*∗*^ for the whole group and by sex.

Type of activity	% whole sample	% male caregivers^*∗*^	% female caregivers^*∗*^
Physical	97	24	73
Social	48	9	39
Home-based entertainment	20	4	16
Daily	30	6	24
Total N	195	43	152

^*∗*^Participation weighted % according to sample size.

**Table 4:** PDQ-Carer Questionnaire for the whole sample and by sex.

PDQ-carer factor	Whole sample				Male caregivers				Female caregivers			
	Mean	SD	N QoL compromised^*∗*^	% QoL compromised	Mean	SD	N QoL compromised	% QoL compromised relative to male sample	Mean	SD	N QoL compromised	% QoL compromised relative to female sample
Personal and social activities	43.64	20.12	19	25	28.13	18.34	1	6	47.85	18.56	18	31
Anxiety and depression	42.33	22.53	15	20	30.73	18.87	1	6	45.48	22.55	14	24
Self-care	44.00	25.27	23	31	32.50	23.81	2	13	47.12	24.94	21	36
Stress	51.06	26.82	34	45	35.68	22.46	3	19	55.23	26.54	31	53

## Data Availability

The data used to support the findings of this study are available from the corresponding author upon request.

## References

[1] Fearnley J. M., Lees A. J. (1991). Ageing and Parkinson’s disease: substantia nigra regional selectivity. *Brain*.

[2] Lotharius J., Brundin P. (2002). Pathogenesis of Parkinson’s disease: dopamine, vesicles and *α*-synuclein. *Nature Reviews Neuroscience*.

[3] Jankovic J. (2008). Parkinson’s disease: clinical features and diagnosis. *Journal of Neurology, Neurosurgery & Psychiatry*.

[4] Poewe W. (2008). Non-motor symptoms in Parkinson’s disease. *European Journal of Neurology*.

[5] Chaudhuri K. R., Schapira A. H. (2009). Non-motor symptoms of Parkinson’s disease: dopaminergic pathophysiology and treatment. *The Lancet Neurology*.

[6] White N. E., Richter J. M., Fry C. (1992). Coping, social support, and adaptation to chronic illness. *Western Journal of Nursing Research*.

[7] Goy E. R., Carter J. H., Ganzini L. (2008). Needs and experiences of caregivers for family members dying with Parkinson disease. *Journal of Palliative Care*.

[8] Wressle E., Engstrand C., Granérus A.-K. (2007). Living with Parkinson’s disease: elderly patients? and relatives? perspective on daily living. *Australian Occupational Therapy Journal*.

[9] Leroi I., Harbishettar V., Andrews M., McDonald K., Byrne E. J., Burns A. (2012). Carer burden in apathy and impulse control disorders in Parkinson’s disease. *International Journal of Geriatric Psychiatry*.

[10] Caap-Ahlgren M., Dehlin O. (2002). Factors of importance to the caregiver burden experienced by family caregivers of Parkinson’s disease patients. *Aging Clinical and Experimental Research*.

[11] McLaughlin D., Hasson F., Kernohan W. G. (2011). Living and coping with Parkinson’s disease: perceptions of informal carers. *Palliative Medicine*.

[12] Soh S.-E., Morris M. E., McGinley J. L. (2011). Determinants of health-related quality of life in Parkinson’s disease: a systematic review. *Parkinsonism & Related Disorders*.

[13] de Dreu M. J., Kwakkel G., van Wegen E. E. H. (2015). Partnered dancing to improve mobility for people with Parkinson’s disease. *Frontiers in Neuroscience*.

[14] dos Santos Delabary M., Komeroski I. G., Monteiro E. P., Costa R. R., Haas A. N. (2018). Effects of dance practice on functional mobility, motor symptoms and quality of life in people with Parkinson’s disease: a systematic review with meta-analysis. *Aging Clinical and Experimental Research*.

[15] Hackney M., Earhart G. (2009). Effects of dance on movement control in Parkinson’s disease: a comparison of Argentine tango and American ballroom. *Journal of Rehabilitation Medicine*.

[16] Pacchetti C., Mancini F., Aglieri R., Fundarò C., Martignoni E., Nappi G. (2000). Active music therapy in Parkinson’s disease: an integrative method for motor and emotional rehabilitation. *Psychosomatic Medicine*.

[17] Song R., Grabowska W., Park M. (2017). The impact of Tai Chi and Qigong mind-body exercises on motor and non-motor function and quality of life in Parkinson’s disease: a systematic review and meta-analysis. *Parkinsonism & Related Disorders*.

[18] Sudarsky I., Bernazzoli B., Costantino C. (2017). Systematic review on strength training in Parkinson’s disease: an unsolved question. *Clinical Interventions in Aging*.

[19] Stegemöller E. L., Radig H., Hibbing P., Wingate J., Sapienza C. (2017). Effects of singing on voice, respiratory control and quality of life in persons with Parkinson's disease. *Disability and Rehabilitation*.

[20] Martikainen P., Bartley M., Lahelma E. (2002). Psychosocial determinants of health in social epidemiology. *International Journal of Epidemiology*.

[21] Lewis C., Annett L. E., Davenport S., Hall A. A., Lovatt P. (2014). Mood changes following social dance sessions in people with Parkinson’s disease. *Journal of Health Psychology*.

[22] McGill A., Houston S., Lee R. Y. W. (2014). Dance for Parkinson’s: a new framework for research on its physical, mental, emotional, and social benefits. *Complementary Therapies in Medicine*.

[23] Shanahan J., Morris M. E., Bhriain O. N., Saunders J., Clifford A. M. (2015). Dance for people with Parkinson disease: what is the evidence telling us?. *Archives of Physical Medicine and Rehabilitation*.

[24] Shanahan J., Morris M. E., Bhriain O. N., Volpe D., Lynch T., Clifford A. M. (2017). Dancing for Parkinson disease: a randomized trial of Irish set dancing compared with usual care. *Archives of Physical Medicine and Rehabilitation*.

[25] Martire L. M., Lustig A. P., Schulz R., Miller G. E., Helgeson V. S. (2004). Is it beneficial to involve a family member? A meta-analysis of psychosocial interventions for chronic illness. *Health Psychology*.

[26] Whetten-Goldstein K., Sloan F., Kulas E., Cutson T., Schenkman M. (1997). The burden of Parkinson’s disease on society, family, and the individual. *Journal of the American Geriatrics Society*.

[27] Carter J. H., Stewart B. J., Archbold P. G. (1998). Living with a person who has Parkinson’s disease: the Spouse’s perspective by stage of disease. *Movement Disorders*.

[28] Trend P., Kaye J., Gage H., Owen C., Wade D. (2002). Short-term effectiveness of intensive multidisciplinary rehabilitation for people with Parkinson’s disease and their carers. *Clinical Rehabilitation*.

[29] Simons G., Thompson S. B. N., Smith Pasqualini M. C. (2006). An innovative education programme for people with Parkinson’s disease and their carers. *Parkinsonism & Related Disorders*.

[30] Marziali E., Donahue P., Crossin G. (2005). Caring for others: internet health care support intervention for family caregivers of persons with alzheimer’s, stroke, or Parkinson’s disease. *Families in Society: The Journal of Contemporary Social Services*.

[31] Secker D. L., Brown R. G. (2005). Cognitive behavioural therapy (CBT) for carers of patients with Parkinson’s disease: a preliminary randomised controlled trial. *Journal of Neurology, Neurosurgery & Psychiatry*.

[32] Martínez-Martín P. (1995). An Quality of life in Parkinson’s disease: validation study of the PDQ-39 Spanish version. *Journal of Neurology*.

[33] Ryff C. D., Lee C., Keyes M. (1995). The structure of psychological well-being revisited. *Journal of Personality and Social Psychology*.

[34] Creswell J. W., Clark V. L. P. (2007). *Designing and Conducting Mixed Methods Research*.

[35] Glaser B. G., Strauss A. L., Glaser B. G., Strauss A. L. (2019). The discovery of grounded theory. *The Discovery of Grounded Theory*.

[36] Patton M. Q. (2002). *Qualitative Research and Evaluation Methods*.

[37] Charmaz K. (2014). *Constructing Grounded Theory*.

[38] Mills J., Bonner A., Francis K. (2006). The development of constructivist grounded theory. *International Journal of Qualitative Methods*.

[39] Corbin J. M., Strauss A. (1990). Grounded theory research: procedures, canons, and evaluative criteria. *Qualitative Sociology*.

[40] Glaser R., Strauss A. (1967). *The Discovery of Grounded Theory*.

[41] Heritage J., Silverman D. (1997). *Qualitative Research: Theory, Method and Practice*.

[42] Gibbs G. (2007). *Analyzing Qualitative Data*.

[43] Jenkinson C., Dummett S., Kelly L. (2012). The development and validation of a quality of life measure for the carers of people with Parkinson’s disease (the PDQ-Carer). *Parkinsonism & Related Disorders*.

[44] Cholewa J., Rychły A., Rafalska B., Cholewa J. (2016). Quality of life amongst care givers for patients with Parkinson’s disease. *Journal of Physical Education & Health-Social Perspective*.

[45] Tysnes O.-B., Storstein A. (2017). Epidemiology of Parkinson’s disease. *Journal of Neural Transmission*.

[46] Morley D., Dummett S., Peters M. (2012). Factors influencing quality of life in caregivers of people with Parkinson’s disease and implications for clinical guidelines. *Parkinson’s Disease*.

[47] Jenkinson B., Grafström M., Winblad B. (1997). Major strain and coping strategies as reported by family members who care for aged demented relatives. *Journal of Advanced Nursing*.

[48] Roland K. P., Chappell N. L. (2015). A typology of care-giving across neurodegenerative diseases presenting with dementia. *Ageing & Society*.

[49] Pinquart M., Sorensen S. (2006). Gender differences in caregiver stressors, social resources, and health: an updated meta-analysis. *The Journals of Gerontology Series B: Psychological Sciences and Social Sciences*.

[50] Barusch A. S., Spaid W. M. (1989). Gender differences in caregiving: why do wives report greater burden?. *The Gerontologist*.

[51] Stoller E. P. (1990). Males as helpers: the role of sons, relatives, and friends. *The Gerontologist*.

[52] Yee J. L., Schulz R. (2000). Gender differences in psychiatric morbidity among family caregivers. *The Gerontologist*.

[53] Park J., Tolea M. I., Arcay V., Lopes Y., Galvin J. E. (2019). Self-efficacy and social support for psychological well-being of family caregivers of care recipients with dementia with Lewy bodies, Parkinson’s disease dementia, or Alzheimer’s disease. *Social Work in Mental Health*.

[54] Lethin C., Renom-Guiteras A., Zwakhalen S. (2017). Psychological well-being over time among informal caregivers caring for persons with dementia living at home. *Aging & Mental Health*.

[55] Challis M. M., Yahr M. D. (1967). Parkinsonism: onset, progression, and mortality. *Neurology*.

[56] Schwab R. S., England A. C., Gillingham F. J., Donaldson I. M. L. (1969). Projection technique for evaluating surgery in Parkinson’s disease. *Third Symposium on in Parkinson’s Disease*.

[57] Buunk B. P., Collins R. L., Taylor S. E., Vanyperen N. W., Dakof G. A. (1990). The affective consequences of social comparison: either direction has its ups and downs. *Journal of Personality and Social Psychology*.

[58] Tanaka K., Quadros A. C. d., Santos R. F, Gobbi L. T. B., Gobbi S. (2009). Benefits of physical exercise on executive functions in older people with Parkinson’s disease. *Brain and Cognition*.

[59] Schrag A., Hovris A., Morley D., Quinn N., Jahanshahi M. (2006). Caregiver-burden in Parkinson’s disease is closely associated with psychiatric symptoms, falls, and disability. *Parkinsonism & related disorders*.

[60] Goldsworthy B., Knowles S. (2008). Caregiving for Parkinson’s disease patients: an exploration of a stress-appraisal model for quality of life and burden. *The Journals of Gerontology Series B: Psychological Sciences and Social Sciences*.

[61] Portillo M. C. (2015). Health care in challenging times of transition. *Journal of Community and Public Health*.

